# Acute *ex vivo* changes in brain white matter diffusion tensor metrics

**DOI:** 10.1371/journal.pone.0223211

**Published:** 2019-09-26

**Authors:** Matthew R. Walker, Jidan Zhong, Adam C. Waspe, Thomas Looi, Karolina Piorkowska, James M. Drake, Mojgan Hodaie

**Affiliations:** 1 Institute of Medical Science, University of Toronto, Toronto, Ontario, Canada; 2 Division of Brain, Imaging and Behaviour - Systems Neuroscience, Krembil Research Institute, University Health Network, Toronto, Ontario, Canada; 3 Centre for Image Guided Innovation and Therapeutic Intervention, Hospital for Sick Children, Toronto, Ontario, Canada; 4 Department of Medical Imaging, University of Toronto, Toronto, Ontario, Canada; 5 Division of Neurosurgery, Hospital for Sick Children, Toronto, Ontario, Canada; 6 Division of Neurosurgery, Toronto Western Hospital, University Health Network, Toronto, Ontario, Canada; Xidian University, CHINA

## Abstract

**Purpose:**

Diffusion magnetic resonance imaging and tractography has an important role in the visualization of brain white matter and assessment of tissue microstructure. There is a lack of correspondence between diffusion metrics of live tissue, ex vivo tissue, and histological findings. The objective of this study is to elucidate this connection by determining the specific diffusion alterations between live and ex vivo brain tissue. This may have an important role in the incorporation of diffusion imaging in ex vivo studies as a complement to histological sectioning as well as investigations of novel neurosurgical techniques.

**Methods:**

This study presents a method of high angular resolution diffusion imaging and tractography of intact and non-fixed ex vivo piglet brains. Most studies involving ex vivo brain specimens have been formalin-fixed or excised from their original biological environment, processes both of which are known to affect diffusion parameters. Thus, non-fixed ex vivo tissue is used. A region-of-interest based analysis of diffusion tensor metrics are compared to in vivo subjects in a selection of major white matter bundles in order to assess the translatability of ex vivo diffusion measurements.

**Results:**

Tractography was successfully achieved in both in vivo and ex vivo groups. No significant differences were found in tract connectivity, average streamline length, or apparent fiber density. Significantly decreased diffusivity (mean, axial, and radial; p<0.0005) in the non-fixed ex vivo group and unaltered fractional anisotropy (p>0.059) between groups were observed.

**Conclusion:**

This study validates the extrapolation of non-fixed fractional anisotropy measurements to live tissue and the potential use of ex vivo tissue for methodological development.

## Introduction

Diffusion magnetic resonance imaging (dMRI) has become widely used to non-invasively study neuroanatomical connectivity and white matter microstructure. Ex vivo specimens can be important tools for dMRI investigations as voxel resolutions on the scale of hundreds of μm can be achieved while in vivo dMRI is limited to voxel sizes in the range of 1–3 mm [1]. The imaging of ex vivo tissue is most frequently performed with fixed samples as these exhibit the longitudinal stability necessary for long scanning times and enhanced voxel resolution. However, fixation is known to alter the diffusion properties of the tissue due to the formation of intermolecular cross-links [2–6]. This suggests that conclusions based on dMRI measurements in fixed tissue may not translate directly to the in vivo environment. Non-fixed ex vivo tissue may be a more appropriate model for some dMRI investigations due to its lack of fixation effects. Thus, we wish to understand the manner in which the diffusion properties of non-fixed ex vivo tissue differs from live tissue and in what contexts the use of ex vivo subjects may be appropriate. This will expand our ability to study both tissue types and translate ex vivo observations and methodological development to in vivo settings.

DMRI is based upon sensitivity to the movement of water in tissue, particularly in white matter where diffusion is anisotropic. Fiber tractography techniques can be used to study connectivity via three-dimensional reconstruction of white matter tracts. Quantitative assessment of selected tracts via diffusion tensor imaging (DTI) metrics can provide insight into white matter microstructure. Fractional anisotropy (FA) is a commonly used measure which broadly describes fiber integrity and is sensitive to local variations in myelination, crossing fiber orientation, and axonal density [7]. Axial (AD) and radial (RD) diffusivities are also widely used metrics which have been correlated with axonal integrity and fiber myelination, respectively [8,9]. Mean diffusivity (MD), an average of all three orthogonal diffusion tensor eigenvalues, reflects the overall degree of water diffusion independent of fiber directionality [10].

Previous studies have reported dMRI examinations of ex vivo tissue in comparison to living tissue including studies of the brain [4,6,11–19], spinal cord [20–22], and excised nerve sections [2,23,24]. Many of these studies involve tissue which has been chemically fixed or extracted from its original biological environment. Others involve tissue which has been affected by trauma [6,13,17] or disorders associated with white matter changes including multiple sclerosis [6,18], alcoholism [4], and stroke [15,19]. As such, their observations with regard to DTI metrics are mixed. In particular, FA has been reported as unchanged [14–16,19] or decreased [6,12,18,21,22,24] in fixed tissue and non-fixed tissue of varying cause of death and scan interval (SI; the time between death and image acquisition) [20,25]. Thus, a measurement of FA without the effects of fixation or other white matter-related changes is needed.

We perform a comparison of the DTI properties of ex vivo brain tissue, extricated from the effects of aldehyde fixation, in order to assess the translatability of scalar diffusion metrics. We also present a method of high angular resolution diffusion imaging and tractography as a guide for region of interest (ROI) placement.

## Materials and methods

### 2.1 Ethics statement

These experiments, including the animal handling and procurement procedures, were approved by the Animal Care Committee at the Hospital for Sick Children in Toronto, ON, Canada and follow the standards set out by the Canadian Council on Animal Care (CCAC).

### 2.2 Subject preparation

**Fig 1:**
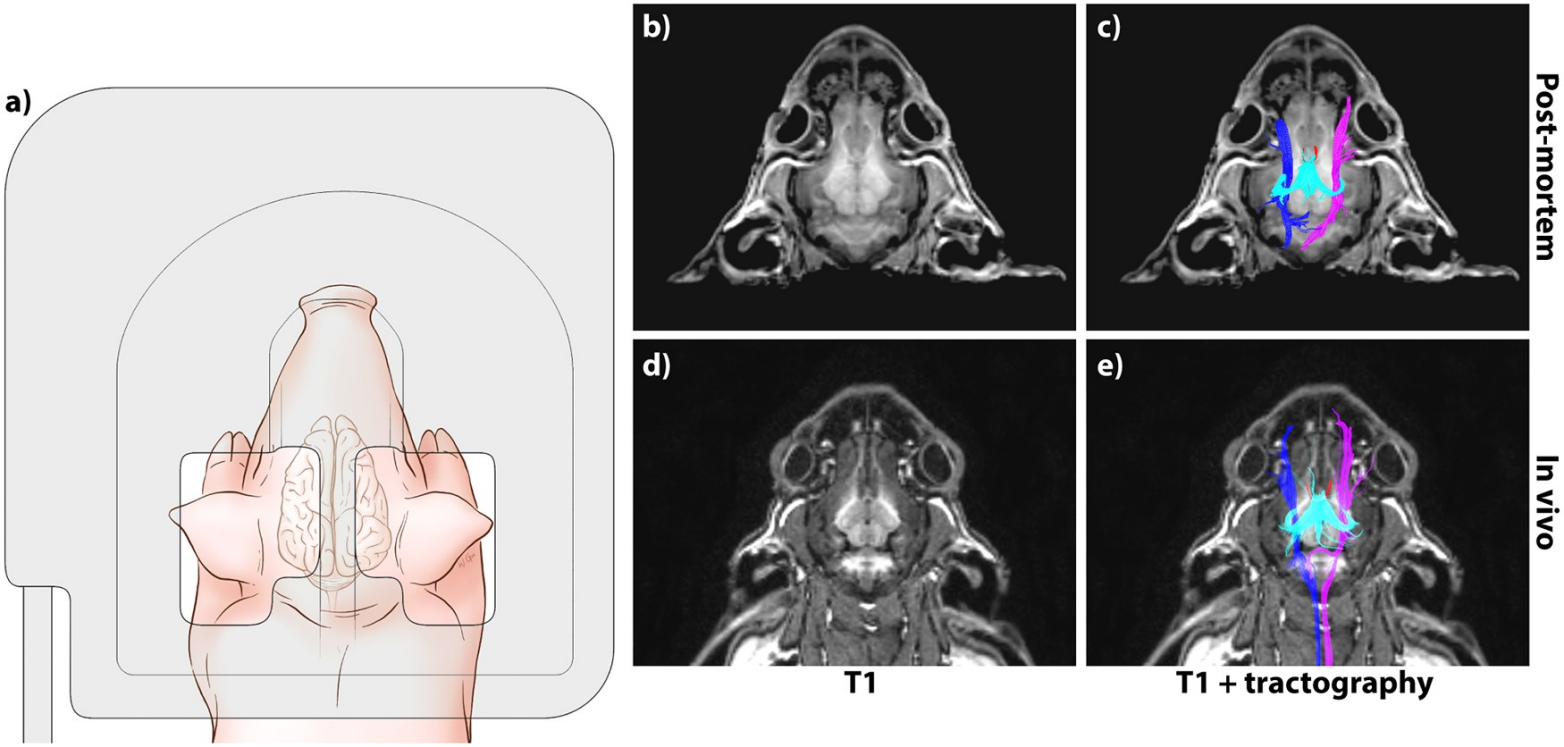
Experimental setup. **a)** Schematic top view of a subject in prone, face first positioning and radiofrequency coil placement. Similar positioning was used for both ex vivo and in vivo subjects. **b,d)** Axial T1 MR images for ex vivo and in vivo subjects, respectively. **c,e)** Axial T1 MR images with overlaid tractography of the fornix (cyan), anterior commissure (red), and left (blue) and right (purple) trigeminal nerves for ex vivo and in vivo subjects, respectively.

Twenty-one male Yorkshire piglets (12 live, 9 ex vivo) were used in this study (LifeTime Solutions Ltd., Ontario, Canada). Animals undergoing in vivo imaging (age: 23±3 days, weight: 6.7±1.1 kg) were housed in a temperature- and humidity-controlled facility with a 12-hour light/dark cycle and fed with commercial piglet milk replacer. Prior to MR scanning, piglets were pre-anesthetized with reconstituted ketamine [11.5 mg/kg IM] (Ketalean, CDMV Quebec, Canada), intubated, and maintained under anesthesia with 2.5% isoflurane and 2 L oxygen supplied by an MR-compatible ventilator system. Piglets were positioned prone and face first in the magnet bore as schematically shown in Fig 1. During acquisition, peripheral capillary oxygen saturation, heart rate, and body temperature were monitored. Maintenance of core body temperature (37°C) was aided via circulating water blanket. At scan completion, piglets were euthanized by intravenous injection of sodium pentobarbital [120 mg/kg IV] (Euthanyl, CDMV Quebec, Canada) while still under general anesthesia.

Nine ex vivo specimens were used in this study, independent from the in vivo cohort (Sumaq Wholesalers Ltd., Ontario, Canada). Researchers were not involved in the sacrifice of ex vivo animal samples. Immediately following euthanasia the piglet heads were removed and cooled in a 4°C refrigerator in order to limit tissue decomposition by autolysis or bacterial degradation [26]. No other surgical or fixation procedures were performed. Prior to imaging, samples were removed from refrigerator and allowed to passively warm in the MR facility. A fiber-optic temperature probe was inserted at the skull base to record sample temperature during image acquisition (18.3 ±2.2°C). Ex vivo subjects were scanned less than 24 hours after death and in the same prone, face first position as the in vivo cohort (Fig 1).

### 2.3 MR image acquisition

Imaging was performed on a 3T scanner (Philips Achieva) using a 32-channel receive-only head coil placed around the subject head, using the same protocol for both subject groups. Three-dimensional anatomical T1-weighted magnetization prepared gradient echo (MPRAGE) images were acquired with the following parameters: repetition time (TR) 8.15 ms; echo time (TE) 3.72 ms; flip angle 8°; matrix 224 x 224; field of view (FOV) 224 mm x 224 mm; slice thickness 1.00 mm; slice number 60; bandwidth 191 Hz/pixel; acquisition duration 14 min 35 s.

High angular resolution diffusion-weighted images (HARDI) were collected using a spin-echo single-shot echo-planar imaging (EPI) sequence with a *b-*value of 800 s/mm^2^ along 128 directions. Additional parameters were: TR 5844.97 ms; TE 105.90 ms; flip angle 90°; matrix 128 x 128; FOV 205 mm x 205 mm; slice thickness 1.60 mm; slice number 38; SENSE reduction factor 2; diffusion gradient pulse duration 15.7 ms, pulse time interval 52.9 ms; bandwidth 1431 Hz/pixel; acquisition duration 29 min 33 s. Additional baseline images with *b*-value = 0 s/mm^2^ were acquired in both forward and reverse phase encoding directions (one in each direction). These baseline images were used in the image post-processing stage to estimate the susceptibility-induced off-resonance fields.

### 2.4 Diffusion analysis and fiber tractography

**Fig 2:**
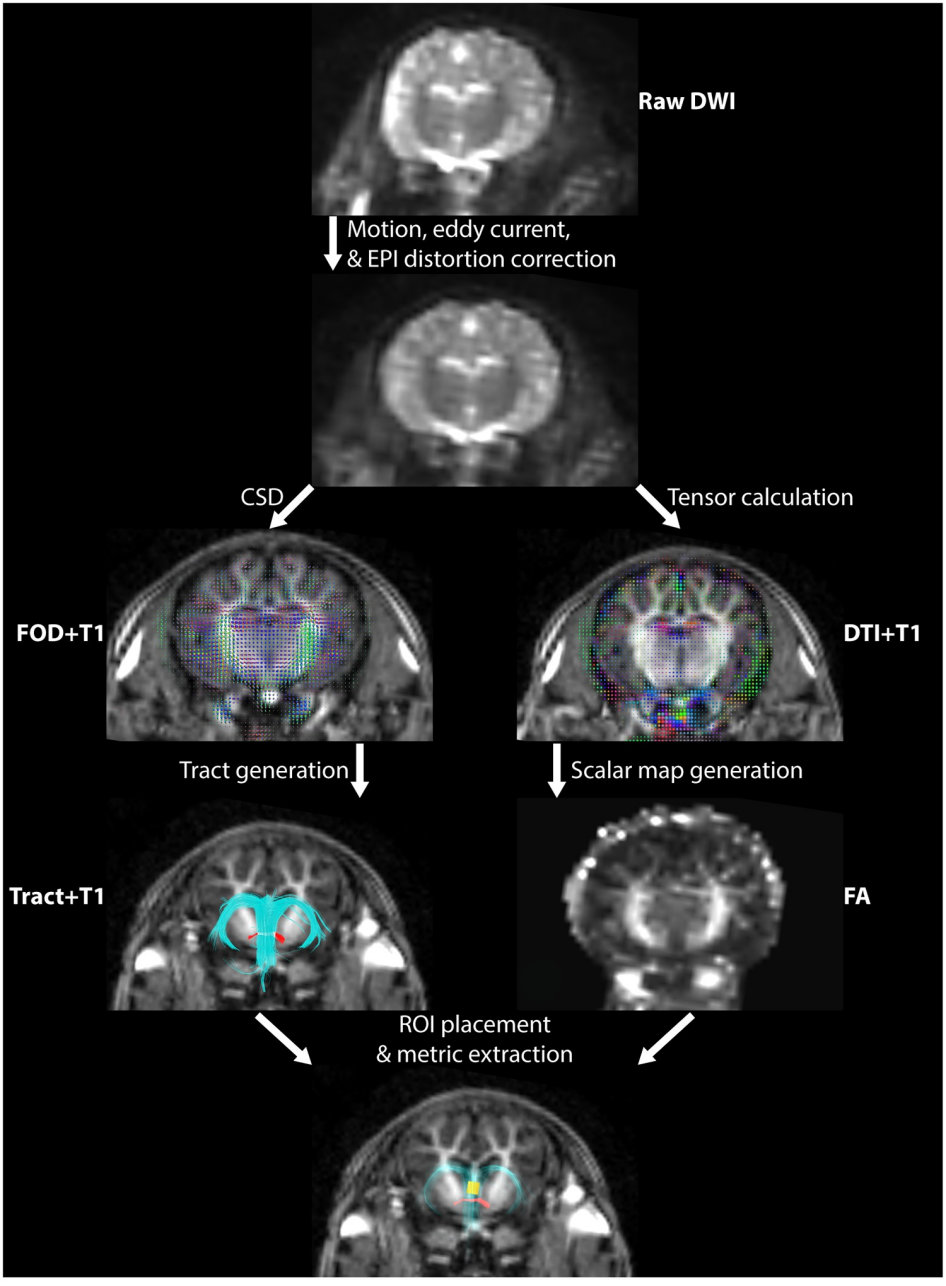
Schematic overview of the diffusion data analysis workflow. Diffusion images are corrected for motion, eddy current, and echo planar imaging (EPI)-based distortions. Corrected data is used to calculate tensors and generate maps of scalar metrics. Fractional anisotropy (FA) map is shown. Corrected data is also used to produce maps of fiber orientation density (FOD) via constrained spherical deconvolution (CSD). FOD and diffusion tensor imaging (DTI) glyphs are shown overlaid with anatomical T1 image. Tractography is generated from FOD map with seeds placed on T1. Tractography guided region of interest (ROI; yellow box) placement for the extraction of scalar metrics.

A schematic overview of the diffusion data analysis is shown in Fig 2. The diffusion-weighted data sets were corrected for distortions caused by EPI and susceptibility-induced off-resonance fields [27] using the forward and reverse phase-encoded baseline images per the method introduced by Chang and Fitzpatrick [28] and implemented in FSL “topup” (Analysis Group, FMRIB, Oxford, UK: https://fsl.fmrib.ox.ac.uk/fsl/fslwiki) [29–31]. The forward and reverse phase-encoded baselines resulted in a pair of images with distortions in opposite directions. In “topup” this pair is used to estimate the underlying susceptibility-induced off-resonance field and the two images with opposing distortions are combined into a single corrected one. This estimated field is then used to remove susceptibility distortions from the entire diffusion data set. Corrections for eddy current-induced distortions and subject movements were performed using “eddy” in FSL [32]. This method is based upon the registration of individual volumes of the diffusion data set to the baseline to account for unique eddy current distortions present in each volume and any subject movement taken place between the acquisition of successive volumes.

Following these distortion corrections, diffusion tensors were calculated using an iteratively reweighted linear least squares estimator [33]. Tensors were then used to generate scalar maps of fractional anisotropy (FA), radial (RD), axial (AD), and mean (MD) diffusivity [34,35]. Co-registration of diffusion and anatomical T1 images was accomplished using the FSL FMRIB Linear Image Registration Tool (FLIRT) [36].

Fiber tracking was performed using MRtrix (Brain Research Institute, Melbourne, VIC, Australia: https://www.florey.edu.au/imaging-software). Non-brain tissue was excluded from fiber tracking using a mask generated from the corrected DWI image [37]. Intensity normalization was performed in order to accurately compare fiber densities for each tract in each group. A single fiber response function for constrained spherical deconvolution was calibrated within this mask using the recursive framework laid out in [38]. Briefly, the response function is an estimation of the signal intensity expected in a given diffusion data set for coherently-oriented fiber bundles. The response function was then used to estimate the fiber orientation distribution (FOD) function [39] using the algorithm described in [40]. In this step, constrained spherical deconvolution (CSD) is performed to model the diffusion signal on a basis of spherical harmonics. This modelling approach has been shown to be well-equipped to handle voxels which contain multiple fiber orientations (such as crossing fibers) [41]. Fiber tracks were generated from the FOD using SD-Stream, a deterministic streamline tracking algorithm [42]. Tracking parameters were: step size 0.16 mm; stopping angle 30°; stopping and initial fiber orientation amplitude 0.1; generated tracks 1000. Seed placement was determined by directionally encoded color (DEC) diffusion maps which were weighted by the FOD and panchromatically sharpened by the higher resolution T1 anatomical image [43,44].

Four major white matter bundles were selected for fiber tracking: corpus callosum, fornix, optic nerve and tract, and trigeminal nerve. These tracts were selected because they cover a range of cortical, subcortical, and brainstem connections and are large, uniquely shaped bundles which can be robustly reconstructed via tractography. Tractography for each respective bundle was generated from a seed mask along the sagittal midline of the corpus callosum and spherical point seeds placed on the posterior body of the fornix, centre of the optic chiasm, and anterior to the pontine cistern in the bilateral trigeminal nerves. Average fiber length and apparent fiber density (the latter obtained by dividing fiber volume by streamline length) were measured for each generated tract and compared across groups. These quantitative tract comparisons, as well as qualitative assessment of anatomical connectivity, serve as quality assurance for the image and tract quality in guiding region of interest (ROI) placement. Significant tractography differences between groups might suggest that these images are not reliable for ROI guidance and metric analysis.

A ROI-based analysis was performed to extract scalar metrics (FA, RD, AD, and MD) from voxels containing fiber projections. Separate ROIs were used for subregions and bilateral projections (for non-midsagittal structures) in order to capture local variability in diffusion metrics. Tractography was used for guidance in ROI placements to provide assurance that metrics are extracted from the selected white matter bundles and minimize infiltration from adjacent grey matter, cerebrospinal fluid, or white matter of non-interest. ROIs were drawn in template space and transformed to individual subject space for metric extraction in order to limit experimenter bias and give equal weight to each subject’s contribution to the group analysis. The corpus callosum was subdivided into five vertical partitions in the anterior-posterior extent as proposed by Hofer and Frahm [45]. The fornix body and column were delineated as reported previously by Chen et al. [46]. The optic nerve and tract were selected from the visual system while the optic chiasm was omitted due to crossing fibers potentially obfuscating the diffusion tensor measurement. Finally, the root entry zone (REZ) and cisternal portions of the trigeminal nerve were examined as previously done by DeSouza et al. [47].

### 2.5 Statistical analysis

Group comparisons of DTI metrics between in vivo and ex vivo ROIs were performed using two-tailed, independent sample *t*-tests with temperature as a covariate. Shapiro-Wilk tests of normality were used to confirm that the data is normally distributed. Levene’s test was used to verify equivalence of variance. For data where the assumption of homogeneity of variances is not satisfied, Welch’s analysis of variance is performed. A Bonferroni correction for multiple comparisons resulted in an adjusted alpha threshold of p<0.003. A power analysis was performed using a confidence level of 5% and statistical power level of 80% resulting in an independent sample size of 9.

## Results

### 3.1 Diffusion metrics

**Fig 3:**
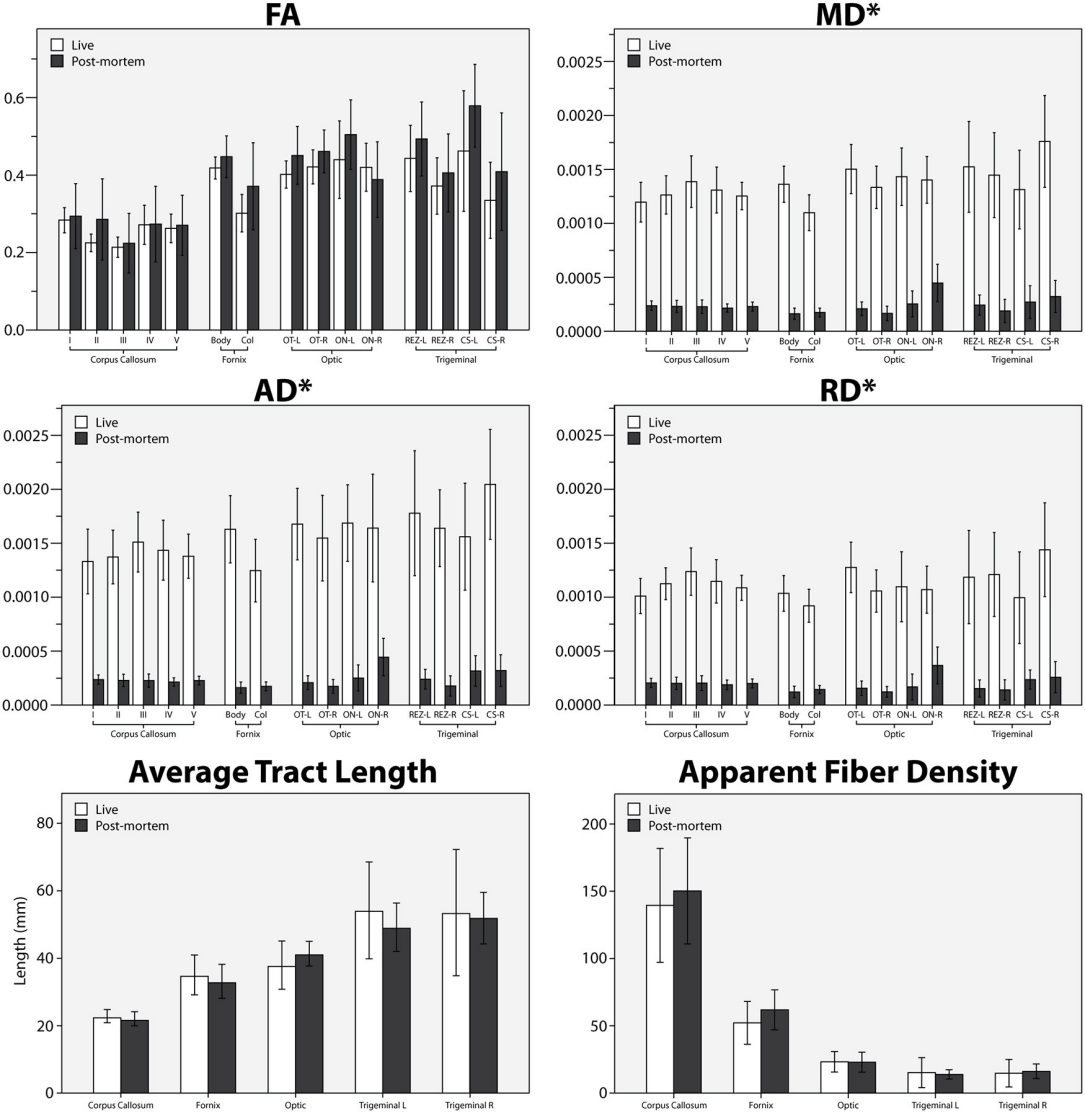
Diffusion tensor imaging (DTI) metric and tract comparisons. DTI metrics of fractional anisotropy (FA) and mean (MD), axial (AD), and radial diffusivity (RD) in rows 1 and 2. Tract comparisons of average tract length and apparent fiber density shown in row 3. Diffusion metric results are grouped by white matter bundle (corpus callosum, fornix, optic nerve and tract, and trigeminal nerve). Subregions for each bundle include the anterior-posterior subdivisions of the corpus callosum (I-V); fornix body and column; optic nerve (ON) and tract (OT) on both left and right sides (L, R); and trigeminal nerve root entry zone (REZ) and cisternal segment (CS) on left and right sides. Tract results are grouped by fiber bundle only due to generation by single seed location. Asterisks indicate significant ex vivo decreases in MD, AD, and RD across all subregions examined (p<0.0005).

Quantitative assessment of diffusion metrics were performed for each ROI across both groups (Fig 3). Significantly lower MD, AD, and RD were observed in all tracts of non-fixed ex vivo brains compared to in vivo (p<0.0005). Decreases ranged from 65% to 88%. The statistical significance of diffusivity reductions is observed in both Welch’s analysis of variance and analysis of covariance with temperature. FA was found to be unaltered (p>0.059); no statistically significant differences were seen between DTI measurements before and after death for any ROI.

Consistent differences in anisotropy were found across the five subregions of the corpus callosum in both in vivo and ex vivo groups. A trend was observed where FA values in the extreme anterior and posterior subregions (I and V, respectively, with values between 0.26 and 0.29) were higher than those in the middle subregions (most notably region III with values of roughly 0.21). This is similar to previous findings of [45] and suggests that the proposed human corpus callosum classification scheme may also appropriate when applied to porcine subjects.

### 3.2 Tractography reconstruction of white matter structures

Visual reconstruction of selected white matter structures was successfully performed in all imaged in vivo and ex vivo piglet brains. Qualitative inspection revealed no differences between groups with regard to anatomically accurate projections or spurious fibers. No bundle in any subject failed to meet the threshold of 1000 generated fiber counts per tract and did not exceed 1152 fiber seed attempts, well below the default maximum attempt number of 100,000. Quantitative comparison showed no significant differences between groups in average tract length or apparent fiber density (Fig 3).

**Fig 4:**
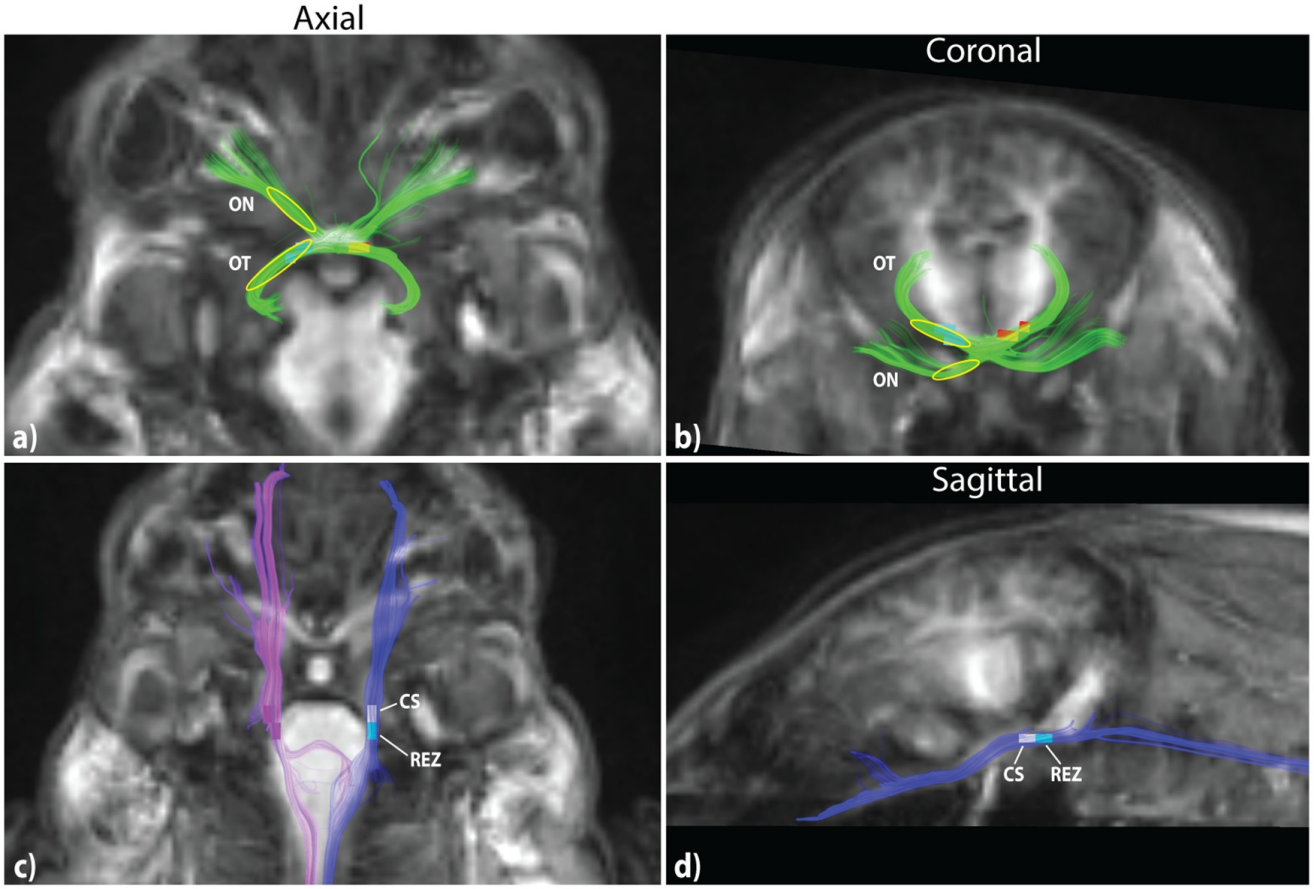
T1-weighted images and fiber tractography of the optic nerves (ON), optic tracts (OT), and trigeminal nerves. Region of interest (ROI) voxels for metric extraction are shown in slices where possible and yellow ellipsoids indicating ROI placement on tracts covering multiple slices. **a,b)** ON and OT (green). **c,d)** Trigeminal nerves (left- blue; right- purple). Regions of interest (ROIs) were placed in the cisternal segments (CS) and root entry zones (REZ) of each nerve.

The optic fibers (Fig 4a and 4b) could be tracked from the anterior portions of the optic chiasm, where the tracking seeds were placed, proceeding posteriorly through the chiasm into the optic tracts and terminating in the lateral geniculate nuclei. A majority of fibers from each optic nerve decussated at the chiasm to the contralateral tract though some fibers were observed projecting ipsilaterally.

The trigeminal nerves (Fig 4c and 4d) were reconstructed bilaterally from the anterior region of the pig face through the pontine cistern and brain stem root entry zone at the belly of the pons. Tracts descended through the brain stem and into the spinal cord. However, given our imaging resolution and the tract diameters, we cannot conclude that the reconstructed fibers are projecting directly into the primary trigeminal nucleus.

**Fig 5:**
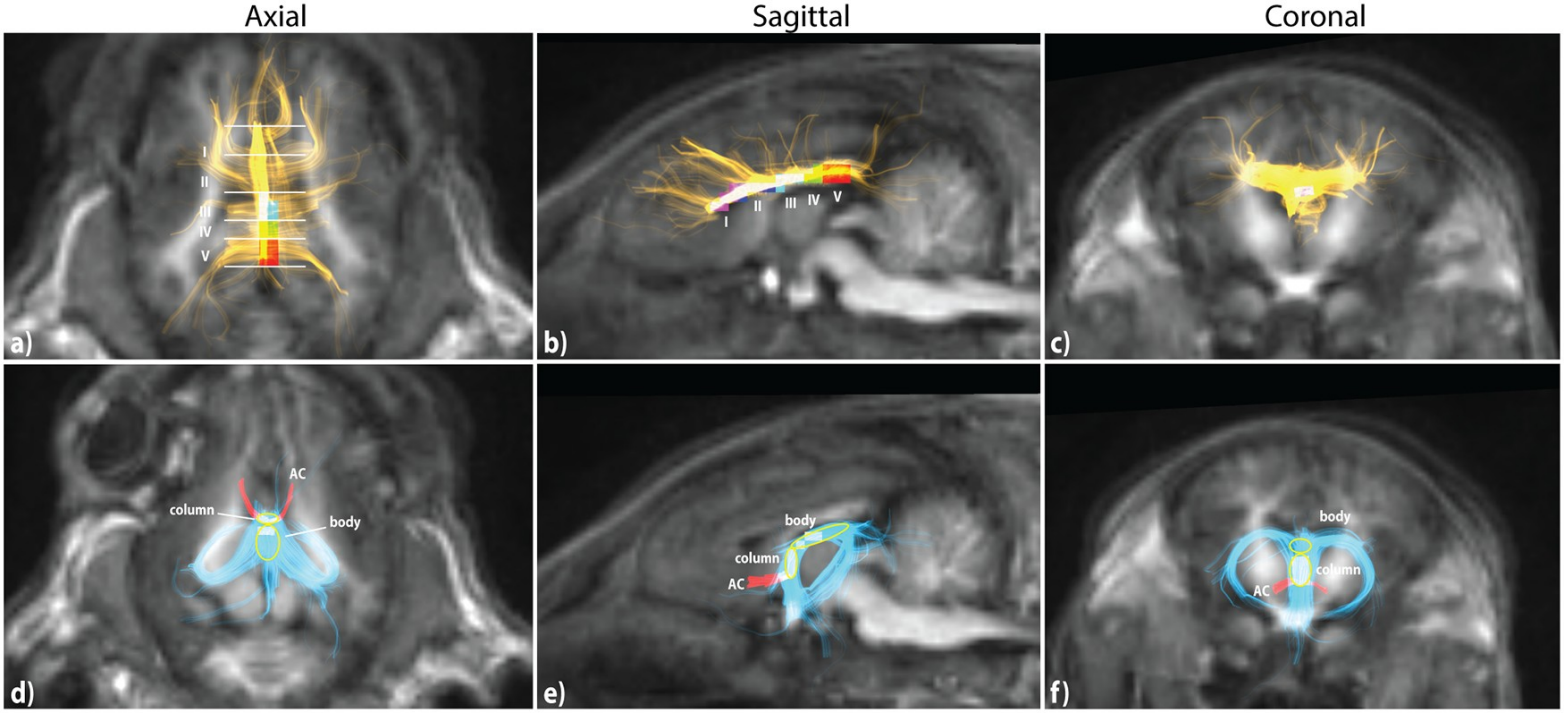
T1-weighted images and fiber tractography of the corpus callosum (yellow), fornix (cyan), and anterior commissure (AC; red). Region of interest (ROI) voxels for metric extraction are shown in axial, sagittal and coronal slices where possible with yellow ellipsoids indicating ROI placement on tracts covering multiple slices. **a-c)** Corpus callosum. Five anterior-posterior subregions (I-V) are defined for ROI placement. **d-f)** Fornix and AC. Midline subregions body and column are identified. AC is used as a reference to define the inferior boundary of the column.

Tracking seeds placed along the median sagittal section of the corpus callosum produced tracts which covered the entirety of the structure from the anterior portion of the genu to the posterior aspect of the splenium (Fig 5a–5c). Connections were observed anteriorly to the prefrontal cortex via the genu, superiorly to the frontal and parietal cortical regions via the body, and laterally to the temporal lobe via the splenium.

Tracts of the body and column of the fornix were well visualized (Fig 5d–5f). The body is defined as the midline structure which courses anteriorly following the roof of the third ventricle. The column is distinguished as the short vertical segment between the anterior fornix body and the anterior commissure (AC). Inferior to the AC, the fornix is comprised of the bilateral pre- and post-commissural columns which travel anterior and posterior to the AC, respectively. Posterior to the body the tracts course bilaterally towards the hippocampal formations via the fornix crura and fimbria.

## Discussion

This study highlights the utility of DTI in ex vivo, non-fixed piglet brains. Specifically, we demonstrate that FA, considered the overall assessment of fiber integrity, is preserved for live and non-fixed ex vivo brain tissue despite a 65–88% reduction in overall water diffusion. Measures of MD, AD, and RD were significantly decreased ex vivo. Robust tractography of a variety of white matter bundles is achievable in both in vivo and ex vivo brains with no significant differences in average tract length or apparent fiber density. Reconstructed tracts were produced using identical diffusion post-processing and tracking methods. This validates the extrapolation of anisotropy measurements in ex vivo animal models to in vivo situations.

### 4.1 Fractional anisotropy remains unaltered ex vivo

Our study demonstrates that FA remained unchanged despite significant reductions to all three diffusion tensor eigenvalues post-mortem. It is inferred that the tensor eigenvalues decrease proportionally in all directions such that the directionality of diffusion is unaffected. The presumed biological components of diffusion anisotropy are the cylindrical axon microtubules and neurofilaments [7]. These structural barriers restrict the motion of water molecules more in some directions than others. Our results suggest that, following death, these barriers persist while the absolute rate of water motion is hindered.

Previous studies have reported mixed findings on whether FA is preserved [14–16,19] or reduced [6,12,18,21,22,24] in formalin-fixed brain and spinal cord tissue. Unaltered FA, as we present here for non-fixed animal brains, has been observed in two studies of non-fixed human tissue in the brain [25] and in peripheral nerves [20]. Ex vivo subjects included in these studies vary in cause of death, scanning interval (SI; the time from death to image acquisition), and MR scanning parameters relative to those used for living participants. Different field strengths and *b*-values may induce differences in diffusion measurements [48,49]. Animals presented in our study were imaged with identical protocols, similar SI (< 24 hours), and consistent euthanization method.

Aldehyde fixatives stabilize tissue metabolically and structurally by cross-linking protein amine groups throughout intra- and extracellular spaces [50]. This temporal stability allows for considerably longer scan times for fixed tissue which can provide improved spatial resolution and SNR. However, fixation may alter the local cellular membranes experienced by water molecules and affect the rate and directionality of water diffusion in those compartments. Further, the fixative compound used and fixation procedure (perfusion or immersion) can affect tissue MR properties differently [2]. This variability in fixation method may explain the lack of consensus in the literature about diffusion anisotropy in fixed tissue and highlight the difficulty of extrapolating ex vivo diffusion measurements to their in vivo counterparts. The data we present here of unaltered FA in non-fixed brain tissue suggests that anisotropy measurements can be translated to the in vivo environment.

### 4.2 Decreased diffusivities

Our diffusivity measurements of MD, AD, and RD are significantly decreased from their in vivo counterparts and thus cannot be translated freely between groups. A substantial factor leading to diminished overall diffusivity is likely the decreased temperature of scanned ex vivo tissues. Le Bihan [51] previously demonstrated that a 2.4% decrease in water diffusivity is to be expected for every 1°C decrease in tissue temperature. Our ex vivo brains were measured to be roughly 19°C less than our in vivo subjects at the time of scanning. Based on Le Bihan’s relation and the temperature measurements of the subjects in this study, we would expect a 45.6% decrease in water diffusivity in our ex vivo tissue. However, we observed a 65–88% decrease in diffusivity. A previous study by Scheurer et al. also reported that diffusivity differences persist when applying temperature corrections in a similar manner [25]. Further, our observed decreases in MD, AD, and RD remained statistically significant when using temperature as a covariate in the analysis. It appears that temperature differences alone between ex vivo and in vivo tissue do not adequately explain the discrepancies in diffusivity measurements.

An additional process affecting our diffusivity observations could involve axonal transport, the process by which cellular organelles are moved to and from the neuron cell body via axon microtubules. Absolute diffusion rates may be aided by this process in a living organism and presumably hindered ex vivo. Axonal transport has been evaluated for its role in anisotropic diffusion in excised garfish nerves that were treated with vinblastine, a substance which depolymerizes microtubules and inhibits axonal transport [23]. The authors found diffusion anisotropy to be unaltered but observed 30–50% decreases in apparent diffusivity coefficient relative to untreated nerves.

Our study agrees with previous observations in both fixed and non-fixed ex vivo nervous tissue where diffusivity is significantly decreased by 30% or more [6,11,12,15,16,18–21,23,25]. A single study examining immersion-fixed rat cortical brain slices demonstrated a 4% increase in apparent diffusion coefficient [2]. However, diffusion results in prepared cortical slices may not readily translate to intact white matter tissue.

### 4.3 Image timing and fixation of ex vivo specimens

Since tissue fixation processes either halt or substantially slow down the metabolic decay process, careful consideration must be taken regarding post-mortem interval (PMI), the time from death to fixation, and SI, the time from death to image acquisition. Previous fixation studies show a heavy correlation of PMI and, to a lesser degree, SI with decreases in FA and diffusivity measures [6,12]. In both cases, but with particular regard to PMI, tissue autolysis and bacterial degradation are given time to occur and may cause changes in tissue microstructure.

These degenerative processes may be mitigated somewhat in non-human research studies where tissues may be fixed via transcardial perfusion shortly after ex vivo status, resulting in small PMI, in addition to immersion fixation following tissue extraction. Cardiac injection may be performed pre-mortem in some cases, resulting in PMI of zero [1]. A process consisting of perfusion fixation followed by immersion and cooled storage has been shown to effectively stop autolytic degeneration processes where diffusion metric measurements were stable for a period of up to three years [52]. It should be noted that, though stable over long periods, the MR properties of the tissue remain subject to the effects of fixation itself.

With regard to human samples, this perfusion fixation approach is generally not possible. In humans, immediate transcardial perfusion at death is not possible, resulting in long PMI and thus more time for tissue degeneration to occur. Often, immersion fixation is the only possible avenue for fixation in human specimens, resulting in poorly fixed tissue due to the longer time scales for fixative compounds to passively diffuse into the tissue from the outside in, effectively increasing the PMI for interior tissue [1].

Because these decay processes are limited by cold temperatures, our ex vivo specimens were stored in a 4°C refrigerator before scanning. A previous study whose authors employed similar sample handling methods found no correlation between FA measurements and SI over a mean 40 hour period in non-fixed human brains [25]. These results and ours suggest that FA measurements in non-fixed brain tissue are preserved for at least 24 hours post-mortem given that the samples are stored in a cooled environment until they are to be scanned, at which point they should be allowed to passively warm to room temperature before scanning. Decay processes will reengage at warmer temperatures, however, which would preclude days-long scanning durations sometimes used with fixed tissue. In this case scanning should be kept to typical clinical scan durations (~one hour) to minimize decay-related tissue changes during acquisition.

### 4.4 Implications for imaging of white matter ex vivo

Our findings do not disqualify the use of fixed tissue samples for studies of tissue cytoarchitecture or white matter connectivity. Rather, we wish to highlight the potential translational value of non-fixed tissue as diffusion imaging becomes more ubiquitous in the study of neural microstructure and connectivity. Indeed, fixed tissue samples offer the advantage of temporal stability for time-dependent improvements in image resolution allowing detailed analysis of structure at the micron scale.

However, diffusion-based investigations of ex vivo tissue may be confounded in their extrapolation to in vivo biological environments by the decision to study fixed or non-fixed tissue and the manner in which fixation was performed. The findings reported in this study indicate that anisotropy measurements and tractography may be translated from non-fixed ex vivo to live tissue. This validates the use of ex vivo animal models for methodological or therapeutic development studies which incorporate diffusion imaging without the need for tissue fixation. FA is often cited as a defining metric in comparisons between pre- and post-surgical assessment and between disease and control groups. A successful surgical treatment may be defined in part by its ability to restore altered FA measurements in a patient to that of a healthy non-patient group. Novel neurosurgical procedures, for example, require thorough characterization before use in living humans. Phantoms or fixed tissue specimens may provide stable imaging but may not be a good model for the structural complexity or physical characteristics of live tissue. In these cases the translatability of such pre-clinical development and image characterization is hindered. Live animal studies are certainly a viable alternative but these models carry significant logistical and financial burden. Ex vivo animal specimens may circumvent these hurdles in lieu of live subjects. The processing methods presented here are identical between groups. However, scanning protocols may need to be modified for ex vivo subjects in order to account for decreased diffusivity measurements, namely by increasing the diffusion gradient *b*-value [1].

### 4.5 Limitations

Unaltered FA was found between both groups which had otherwise not been treated or altered by exogenous forces. We therefore cannot comment on the ability of FA changes resulting from externally applied procedures in one group to translate identically to the other after the same treatment. With regard to tractography, the tract subregions of interest were defined manually and thus comparisons of average tract length and apparent fiber density between live and ex vivo groups apply only to fully generated tracts. Local tractography variations may exist but are averaged over the entire tract. Subregions of the corpus callosum were defined according to cortical projections observed in humans. These subdivisions may not be appropriate for the cortical connectivity in porcine brains. We refer readers to work by Knosche et al. on downstream cortical tracing in pig brains [53].

Acquisition parameters were identical between groups in the present work, which is a limitation due to the significantly decreased diffusivity observed and its related detrimental effects on SNR. It is suggested that researchers select an appropriately increased b-value for ex vivo scanning (relative to in vivo) to account for decreased diffusivity and signal intensity observed. We refer readers to a recent review article by Roebroeck et al. for a discussion on appropriate scanning and tissue preparation protocols in ex vivo imaging [1].

## Conclusion

The ex vivo status of tissue results in important changes in the diffusion MRI properties and microstructure of the tissue. These changes can be further distinguished between non-fixed tissue and aldehyde-fixed tissue where some diffusion measurements may be preserved in the former but may not be in the latter. Researchers may thus find value in the use of non-fixed specimens to optimize procedures and treatment parameters with an understanding of the translatability of diffusion anisotropy observations to in vivo subjects.
